# Collection of autologous CD34+ hematopoietic progenitor cells (HPC) in multiple myeloma: CD34 + cell collection yield in relation to molecular subtype, karyotype, and FISH results

**DOI:** 10.1371/journal.pone.0349212

**Published:** 2026-05-12

**Authors:** Anwar Rjoop, Ivette Perez, Daoud O. Al Aruri, Majd M. AlBarakat, Frits van Rhee, Gina Drobena

**Affiliations:** 1 Department of Pathology and Microbiology, Jordan University of Science and Technology, Irbid, Jordan; 2 Department of Pathology, University of Arkansas for Medical Sciences, Little Rock, Arkansas, United States of America; 3 Department of General Surgery, Jordan University Hospital, The University of Jordan, Amman, Jordan; 4 Faculty of Medicine, Jordan University of Science and Technology, Irbid, Jordan; 5 Department of Internal Medicine, University of Arkansas for Medical Sciences, Little Rock, Arkansas, United States of America; European Institute of Oncology, ITALY

## Abstract

**Background:**

Autologous stem cell transplantation remains a cornerstone of treatment for patients with transplant-eligible multiple myeloma (MM). Previous studies using gene expression profiling (GEP) have classified MM into seven molecular subgroups: HY, CD-1, CD-2, LB, PR, MS, and MF. Together with GEP signature, it can help identify patients with better or worse outcomes. In this study, we wanted to study how these molecular subtypes, along with cytogenetics, FISH results, and different treatment regimens, relate to the count of CD34 + hematopoietic progenitor cells (HPC) collected during stem cell mobilization.

**Methods:**

We conducted a retrospective study of 505 MM patients who underwent hematopoietic progenitor cell (HPC) collection between January 2017 and December 2020. Demographic, molecular (FISH, cytogenetics, GEP), clinical, treatment, and mobilization regimen data were collected and analyzed to determine factors influencing CD34 + HPC yield.

**Results:**

Factors associated with reduced CD34 + HPC collection included PR and MF GEP subtypes, deletion 13 (−13), and high-risk genetic classification. In contrast, normal versus abnormal karyotype, hyperdiploid versus hypodiploid karyotype, and the use of either proteasome inhibitors in combination with heterogeneous chemotherapy regimens did not significantly impact CD34 + HPC yield.

## 1. Introduction

Multiple myeloma (MM) is a plasma cell neoplasm arising from a clonal expansion of plasma cells in the bone marrow. It is usually associated with considerable morbidity and mortality [1]. The clinical presentation is heterogenous, as it may include bone disease, renal dysfunction, anemia, and hypercalcemia. These manifestations largely reflect bone marrow infiltration and monoclonal production of proteins [2]. Advances in MM biology research have significantly improved treatment options. High-dose chemotherapy followed by autologous stem cell transplantation (ASCT) has remained an important arm of treatment for most patients [3]. ASCT is associated with significant improvements in both progression-free and overall survival in patients with newly diagnosed multiple myeloma (NDMM) [4]. However, successful autologous stem cell transplantation depends on the ability to mobilize and collect an adequate number of CD34 + HPC hematopoietic progenitor cells (HPCs).

Higher CD34 + HPC doses at the time of transplant have generally been associated with more rapid hematologic recovery and fewer early complications following transplantation [4]. On the other hand, stem cell mobilization is not uniform among all patients. A proportion of them have limitations in achieving adequate collections.

This variability suggests that there are some factors related to the patient or specific to the disease that may affect CD34 + HPC yield, for example internal genetic anomalies within the bone marrow microenvironment, underscoring the necessity for further research [4].

Cytogenetic evaluation using conventional karyotyping and fluorescence in situ hybridization (FISH) remains central to risk stratification in MM [5]. Specific abnormalities such as t(4;14), t(14;16), and del(17p) are well-established markers of high-risk disease and are generally associated with worse outcomes [5]. However, the effect of these genetic features on stem cell mobilization and collection is less clear, and existing data have been inconsistent.

This uncertainty persists despite the incorporation of agents, e.g., bortezomib and carfilzomib, which have markedly improved clinical outcomes in multiple myeloma. However, their impact on mobilization outcome, including patients with high-risk cytogenetics, is still not well studied [5].

In this study, we examined the correlation between molecular genetic abnormalities and CD34 + HPC collection in patients with MM. We also assessed the effect of different induction regimens on CD34 + HPC yield prior to ASCT. This study delineates the treatment of multiple myeloma from 2017 to 2020, prior to the widespread adoption of anti-CD38–based quadruplet regimens. Since then, new standards of care, such as using daratumumab-based combinations as the first line of treatment, have had a huge effect on how treatments are done and how effective they are [4,6]. As a result, our findings can be utilized to compare HPC collection outcomes across historical treatment frameworks and more advanced treatment options. To our knowledge our work is the first large-scale analysis (including 505 patients) linking CD34 + HPC collection yield with molecular subtypes (GEP) and detailed cytogenetic abnormalities simultaneously.

## 2. Methods

We conducted a retrospective study of patients newly diagnosed with multiple myeloma who underwent hematopoietic progenitor cell (HPC) collection at a specialized myeloma center at University of Arkansas for Medical sciences between January 2017 and December 2020. A total of 505 patients were included. Data collected included demographics, relevant laboratory data, flow cytometry findings, and molecular studies, including FISH, conventional cytogenetics, and gene expression profiling (GEP). Additional data included the mobilization protocols used for stem cell collection.

The study was approved by the Institutional Review Board of the University of Arkansas for Medical Sciences (IRB approval number 261521; dated August 31, 2020). All analyses were performed in accordance with the principles of the Declaration of Helsinki. No identifiable patient information was used, and study findings did not influence clinical care or treatment decisions.

### 2.1. Study population

This study included all patients who underwent HPC apheresis collection at The University of Arkansas for Medical Sciences between 2017 and 2020. Patient characteristics are shown in Table 1. Eligible participants were 18 years of age or older, with a total of 505 patients meeting the inclusion criteria (209 females and 296 males). The mean age was 61 years (range: 20–80 years); 232 patients had GEP data, 475 had karyotype data, 505 had ploidy assessed by flow cytometry and 295 had FISH data available (Table 2). Patients classified as genetic high-risk had at least one of the following genetic abnormalities: *del(17), t(14;16), t(14;20), or t(4;14)*, or had a GEP subtype of PR, MF, or MS [7].

**Table 1:** Patients characteristics (n = 505).

Characteristics		Total = 505
Sex	Male	296 (58.6%)
	Female	209 (41.4%)
Age (years)	Median (IQR)	63 (14)
Race	White	386 (76.4%)
	Black or African American	99 (19.6%)
	Asian	5 (1.0%)
	American Indian or Alaskan Native	1 (0.2%)
	Native Hawaiian or Other Pacific Islander	1 (0.2%)
	Other	12 (2.4%)
	Unknown	1 (0.2%)
Ethnicity	Hispanic	11 (2.2%)
	Non-Hispanic	494 (97.8%)
Patient Karyotype	Normal	271 (53.7%)
	Abnormal	204 (40.4%)
	Unknown	30 (5.9%)
Chromosomal ploidy	Hyperdiploid	49 (9.7%)
	Hypodiploid	86 (17.0%)
	Normal	370 (73.3%)
GEP cluster	PR	30 (5.9%)
	MF	18 (3.6%)
	MS	23 (4.6%)
	HY	83 (16.4%)
	LB	22 (4.4%)
	CD-1	16 (3.2%)
	CD-2	40 (7.9%)
	Unknown	273 (54.1%)

**Table 2:** Distribution of patients by CD34 + HPC yield and availability of molecular, karyotype, and FISH data.

CD34 + HPC Yield per kg Range (x10^6^/kg)	Total Count of Patients	Count of Patients with GEP	Count of Patients with Karyotype	Count of Patients with FISH
0-5	24	7	22	10
>5-10	36	13	31	19
>10-20	113	47	105	67
>20	332	165	317	199
Total	505	232	475	295

### 2.2. Statistical analysis

Due to large sample size (n = 505) and approximate normality of residuals in regression models, multiple parametric tests were attempted. The patients’ cohort was grouped based on the collection yield (number of CD34 + HPC cells collected). A t-test was used to assess whether genetic high-risk patients collected fewer CD34 + HPC count compared to other patients. Univariate and multivariate linear regression analyses were conducted to evaluate the association between collection yield and other variables, including molecular subtypes. Because CD34 + HPC yields demonstrated a right-skewed distribution with substantial interindividual variability, quantile regression was additionally performed to evaluate the association between induction regimen and CD34 + HPC yield across clinically relevant points of the outcome distribution. Quantile regression analyses were conducted using SPSS Quantile Regression procedures, with prespecified quantiles at the 25^th^, 50^th^ (median), 75^th^, and 90^th^ percentiles to address non-normal distribution of CD34 + yields. Statistical analyses included t-tests and ANOVA. Given the exploratory nature of subgroup analyses, formal correction was not applied. Results are reported as regression coefficients (β) with 95% confidence intervals (CIs) and two-sided *p*-values. A *p*-value <0.05 was considered statistically significant. All analyses were performed using IBM SPSS Statistics, version 23 (IBM Corp., Armonk, NY, USA).

## 3. Mobilization protocols

HPC were collected after mobilization using one of two protocols: (1) with chemotherapy (multiple regimens) plus granulocyte colony-stimulating growth factor (G-CSF) at 5–8 μg/kg twice a day with plerixafor added if needed to achieve the collection goal (dosed subcutaneously at 240 μg/kg when the estimated glomerular filtration rate exceeded 50 mg/min per 1.73 m2 (cap, 40 000 μg) and 160 μg/kg (cap 27 000 μg) in case of lower glomerular filtration rate; or (2) with growth factors alone (G-CSF + /- plerixafor as above). Collection was started when the minimum predicted collection based on peripheral blood CD34 + HPC count was at least 1 x 10^6^ CD34 + HPC cells/kg, a previously validated predictive formula was used to guide the collection process [8].


$$\text{Predicted CD34}+\text{ HPC yield }(\text{cells}/\text{kg})\text{ per liter processed blood}*\;=\frac{\left(\text{peripheral blood CD34 cells}/\text{L})\;(\text{30}\%†\right)}{\text{body weight in kg}}$$

Peripheral blood CD34 + HPC cells (cells/L): measured pre-apheresis

†CE (collection efficiency): assumed to be 0.30 (30%) based on COBE® Spectra performance

*Multiply value by number of liters processed to calculate number of CD34 cells/kg collected

Body weight (kg): donor body weight

The collection goal was 20 x 10^6^ CD34 + HPC cell/kg for all patients, with at least 2 days of collection required by protocol. Collection was performed using a Spectra Optia as previously described [9].

## 4. Results

The study initially analyzed the distribution of MM patients in four different CD34 + HPC collection yield groups. The majority of patients (66%, 332 of 505) fell into the highest yield group (>20 CD34 + HPC cells x 10^6^/kg). In contrast, the lowest collection yield group (0–5 CD34 + HPC cells x 10^6^/kg) represented 4% (22 of 505) of the patients (Table 2).

### 4.1. Treatment regimens

Patients were grouped into those who received standard chemotherapy, including either Bortezomib or Carfilzomib, and patients who received both (received both agents during treatment (not necessarily concurrently)). (Table 3). The comparison between Bortezomib-based regimen and Carfilzomib-based regimen groups showed no statistically significant difference in CD34 + HPC yields. The mean first collection yield was 16.5 ± 16.2 in the Carfilzomib group and 15.3 ± 11.1 in the Bortezomib group (p = 0.708). Similarly, the final CD34 + HPC yield was 29.9 ± 17.9 for Carfilzomib group and 27.0 ± 14.1 for Bortezomib group (p = 0.526).

**Table 3:** Comparison of CD34 + HPC collection yield according to induction regimen.

Regimen	N	First Collection Yield (×10⁶/kg), Mean ± SD	Final Collection Yield (×10⁶/kg), Mean ± SD
Carfilzomib-based	50	16.5 ± 16.2	29.9 ± 17.9
Bortezomib-based	320	15.3 ± 11.1	27.0 ± 14.1
Both	81	14.4 ± 13.3	26.7 ± 15.1
p-value		0.708	0.526

In multivariable linear regression adjusted for age and sex, induction regimen was not independently associated with CD34 + HPC yield (S1 Table). Compared with bortezomib-based induction, carfilzomib-based induction was associated with a non-significant adjusted mean difference of +0.38 × 10⁶ CD34 + HPC cells/kg for first collection (95% CI, −3.13 to 3.90; *p* = 0.83) and +1.83 × 10⁶ cells/kg for the final collection (95% CI, −2.47 to 6.12; *p* = 0.40).

Quantile regression analyses were additionally conducted to assess regimen effects across the distribution of collection outcomes. At the median (50th percentile), carfilzomib-based induction was not associated with a significant difference in first or final CD34 + HPC collection yields, with p-values of 0.32 and 0.63, respectively. Similarly, no significant associations were observed at higher quantiles, including the 75th and 90th percentiles, which correspond to clinically relevant higher collection targets (S1 Table).

At the lower end of the distribution, however, a statistically significant difference was observed at the 25th percentile for first collection yield, where carfilzomib-based induction was associated with a reduced CD34 + HPC yield compared with bortezomib-based therapy (β = −2.94 × 10⁶/kg; 95% CI −5.73 to −0.15; p = 0.040). This finding was not observed in the final cumulative yield.

### 4.2. Cytogenetics

CD34 + HPC cell collection yields were compared according to patient karyotype. There was no significant difference in first apheresis yield between patients with abnormal versus normal karyotypes (mean: 14.2 vs. 15.0 × 10⁶/kg; *p* = 0.46). Similarly, the final collection yield did not differ between karyotype groups (26.5 vs. 26.4 × 10⁶/kg; *p* = 0.98) (Table 4).

**Table 4:** Comparison of CD34 + HPC collection yield between patients with normal and abnormal karyotypes.

Patient Karyotype	Count of Patients	Mean CD34 + HPC Yield (x 10^6^/kg)	p-value
Abnormal (First Collection)	204	14.2	0.46
Normal (First Collection)	271	15.0	
Abnormal (Total collection)	204	26.5	0.98
Normal (Total collection)	271	26.4	

Further analysis compared ploidy status (Table 5). Patients with hyperdiploid karyotypes had slightly higher mean first collection yields (16.1 × 10⁶/kg) compared with normal (14.7 × 10⁶/kg) and hypodiploid patients (12.7 × 10⁶/kg). However, this difference was not statistically significant (*p* = 0.27). Final cumulative CD34 + HPC yields were similarly higher in hyperdiploid patients (28.0 × 10⁶/kg) versus normal (26.5 × 10⁶/kg) and hypodiploid patients (25.0 × 10⁶/kg), without reaching statistical significance as well (*p* = 0.22).

**Table 5:** Comparison of CD34 + HPC collection yield between hyperdiploid, hypodiploid, and normal karyotypes.

Patient Type	N	Mean CD34 + HPC Yield – First Collection (×10⁶/kg)	Mean CD34 + HPC Yield – Final Cumulative (×10⁶/kg)	p-value (First Collection)	p-value (Final Collection)
Hyperdiploid	49	16.1	28.0		
Hypodiploid	56	12.7	25.0		
Normal	370	14.7	26.5		
				0.272	0.221

### 4.3. GEP Subtypes

**Fig 1:**
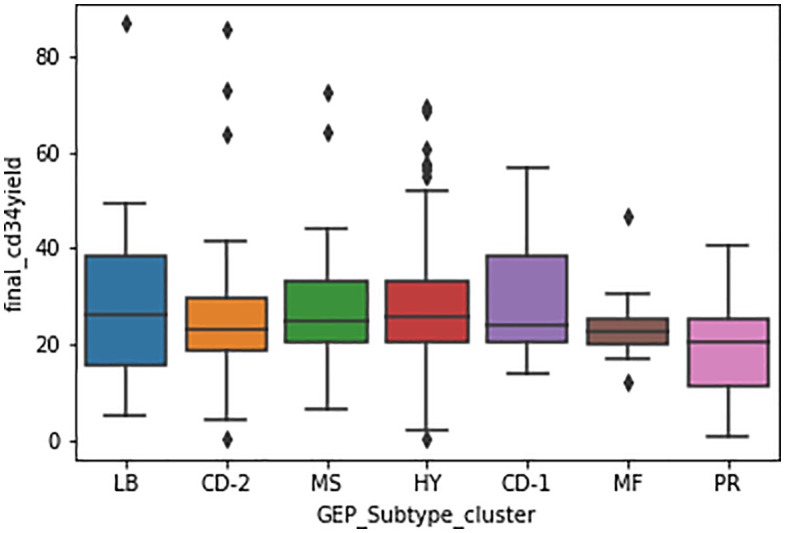
Box plot of total CD34 ⁺ cell collection yield across multiple myeloma GEP subtypes.

Final CD34 + HPC yields were compared across multiple myeloma GEP subtypes (Fig 1). Median yields were broadly similar across LB, MS, HY, and CD-1 subtypes, whereas MF and PR subtypes tended toward lower mobilization. An ANOVA analysis was performed to determine the statistical significance of collection yield among patients with different GEP subtypes. A p-value of 0.06, nearing significance, was detected for at least one GEP subtype.

A two-sample t-test was performed to compare the CD34 + HPC collection yield of patients with the PR subtype against all other patients (Table 6). PR patients had a significantly lower final CD34 + HPC collection yield, with a mean of 19.2 × 10⁶ CD34 + HPC cells/kg compared with 28.0 × 10⁶ CD34 + HPC cells/kg in non-PR patients (p < 0.001). Analysis of the first collection yields also showed a significantly lower count in PR patients, with a mean yield of 10.2 × 10⁶ CD34 + HPC cells/kg versus 15.6 × 10⁶ CD34 + HPC cells/kg in non-PR patients (p = 0.007). A separate t-test comparing patients with the MF subtype to all non-MF patients demonstrated a similar result (Table 6). MF patients had a significantly lower final CD34 + HPC yield (23.9 × 10⁶/kg vs. 28.4 × 10⁶/kg; p = 0.038). First collection yields were also less in MF patients, with a mean of 9.8 × 10⁶ CD34 + HPC cells/kg compared with 16.2 × 10⁶ CD34 + HPC cells/kg in non-MF patients (p = 0.001).

**Table 6:** Association between GEP subtypes (PR and MF) and CD34 + HPC collection yield.

GEP Subtype	Mean CD34 Yield – First Collection – (x 10^6^/kg)	p-value (First Collection)	Mean CD34 Yield – Final collection – (x 10^6^/kg)	p-value (Final Collection)
PR	10.2	0.0068	19.2	0.0001
Non-PR	15.6		28.0	
MF	9.8	0.001	23.9	0.038
Non-MF	16.2		28.4	

### 4.4. Fluorescence in Situ Hybridization (FISH) analysis

S2 Table presents the effect of common chromosomal abnormalities identified by FISH on CD34 + HPC yield. Patients with *deletion of chromosome 13 (−13)* had a significantly lower final CD34 + HPC yield compared with those without the deletion (mean 23.98 × 10⁶/kg vs. 28.55 × 10⁶/kg; p = 0.02). However, the difference observed on the first collection did not meet statistical significance (p = 0.08).

In the case of *t(14;16)(q32;q23)* translocation, patients with this abnormality had significantly lower CD34 + HPC yields on the first collection (mean 6.23 × 10⁶/kg vs. 14.43 × 10⁶/kg; p = 0.03). However, total CD34 + HPC yield was similar between groups (p = 0.85).

*Deletion of chromosome 17 (−17)* analysis did not reach statistical significance for either first-day or total yield (p = 0.14 and p = 0.12, respectively). Although patients with this abnormality demonstrated a consistent trend towards lower CD34 + HPC collection, interpretation is limited by the small number of affected patients (n = 9).

*Amplification of 1q21 (amp(1q21))* and *gain of 14q32 (+14q32)* were both associated with significantly reduced total CD34 + HPC yields (p = 0.04 and p = 0.004, respectively). However, neither mutation showed a statistically significant difference in first collection yield.

### 4.5. Genetic high-risk patients

**Fig 2:**
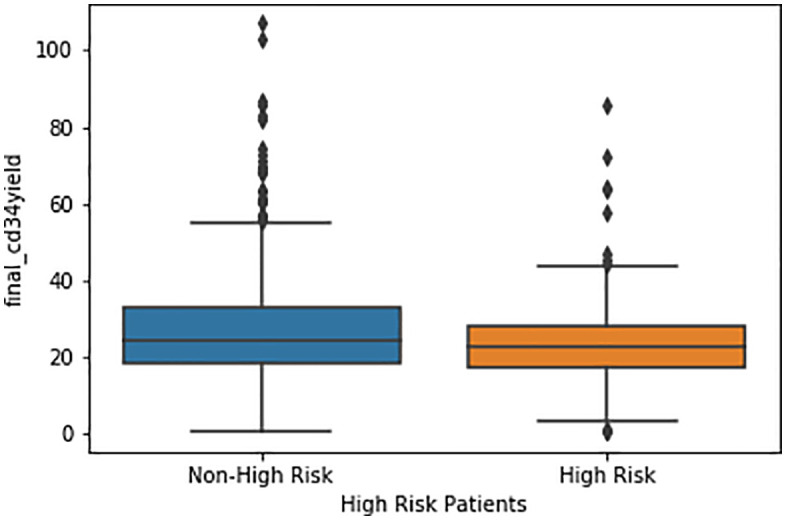
Box-and-whisker plot of final CD34 + cell collection yield (×10⁶/kg) comparing high-risk and non–high-risk patients.

A two-sample t-test was performed to evaluate if high-risk patients collected fewer CD34 + HPC count compared to non–high-risk patients. For the total CD34 + HPC collection yield, high-risk patients had a significantly lower mean yield of 23.9 × 10⁶ CD34 + HPC cells/kg compared with 27.2 × 10⁶ CD34 + HPC cells/kg in non–high-risk patients (p = 0.029). This finding demonstrates a statistically significant reduction in overall HPC collection among high-risk patients (Table 7, Fig 2).

**Table 7:** Comparison of CD34 + HPC HPC collection yield between high-risk and non–high-risk multiple myeloma patients.

Yield Type	Group	Mean CD34 Yield (x 10^6^/kg)	p-value
Final CD34 + HPC Yield	High-Risk Patients	23.9	0.029
	Non-High-Risk Patients	27.2	
First CD34 + HPC Yield	High-Risk Patients	12.8	0.04
	Non-High-Risk Patients	15.3	

Similarly, analysis of the first collection yields showed that high-risk patients collected significantly fewer CD34 + HPC count on the first day of apheresis. The mean collection yield was 12.8 × 10⁶ CD34 + HPC cells/kg for high-risk patients versus 15.3 × 10⁶ CD34 + HPC cells/kg for non–high-risk patients (p = 0.04), indicating impaired early mobilization in the high-risk group (Table 7).

## 5. Discussion

High-dose chemotherapy followed by ASCT has remained a key component of therapy in MM. Many factors influencing HPC mobilization have been described, including patient characteristics, prior therapies, and mobilization strategies themselves [10–15]. In contrast, the role of underlying genetic factors in stem cell mobilization and variability in hematopoietic progenitor cell collection remains poorly defined.

The University of Arkansas for Medical Sciences developed a GEP-based classification system to better understand the biological basis of MM, improve risk stratification, and guide treatment decisions [16]. This system identified seven molecular subgroups that are characterized by the overexpression of distinct gene sets. These subgroups included CD-1, CD-2, MS, MF, PR, LB, and HY. However, this framework has not been shown to predict stem cell collection outcomes. To our knowledge, this study is the first large-scale analysis to examine the effect of individual genetic abnormalities on HPC collection in patients with MM.

### 5.1. Impact of genetic abnormalities on hematopoietic progenitor cell collection

Successful ASCT depends on the ability to collect an adequate number of CD34 + HPC progenitor cells, as higher CD34 + HPC yields have been linked to faster recovery, fewer infectious complications, and improved survival after transplant [17,18]. This underscores the fact that understanding factors that limit CD34 + HPC yield is important for transplant planning.

Conventional metaphase karyotyping has long been used in MM to detect chromosomal abnormalities [8,19]. Despite its limitations, karyotyping remains useful for distinguishing hypodiploid from hyperdiploid disease. In our cohort, we did not observe a significant difference in CD34 + HPC collection between patients with normal and those with abnormal karyotypes (*p* = 0.98). This finding was of particular interest given the well-established association between hypodiploidy and worse clinical outcomes [20]. Our analysis suggests that the worse prognosis associated with hypodiploidy is unlikely to be explained by impaired stem cell mobilization alone and may instead reflect other disease-related factors.

After the adoption of interphase FISH, cytogenetic evaluation of MM has become more informative [21]. This has led to the identification of several high-risk abnormalities associated with inferior survival, including *t(4;14), t(14;16), t(14;20), and del(17p)* [22]. The relationship between cytogenetic risk and stem cell mobilization remains an area of ongoing debate. There is relatively limited and sometimes conflicting data available. For example, Basci et al. evaluated mobilization outcomes in 150 MM patients and found no significant difference between those with normal cytogenetics and those with cytogenetic abnormalities, including high-risk features [23].

Our study examined individual genetic abnormalities rather than grouping them together, allowing for a more detailed assessment of their effects. Other studies have reported mixed results regarding the prognostic significance of these abnormalities on stem cell yields; Lebel et al. observed inferior progression-free survival among patients with very high CD34 + HPC collections (“mega-mobilizers”) and suggested that enhanced mobilization does not necessarily translate into improved long-term outcomes [24]. In contrast, additional reports have linked higher CD34 + HPC yields with better survival [25]. To better assess how cytogenetic abnormalities influence stem cell collection, we analyzed the sixteen most common FISH abnormalities present in our cohort. The majority did not have a significant impact on total CD34 + HPC yield, which again highlights the complexity of MM biology, and conveys that other factors, in addition to high-risk cytogenetics, may play a role in decreased stem cell mobilization in these patients.

### 5.2. Deletion of chromosome 13 and impaired stem cell mobilization

Our analysis confirmed that *deletion of chromosome 13 (−13)* may affect cumulative mobilization rather than initial collection yield. However, it had the most consistent and clinically relevant association with reduced CD34 + HPC collection, supporting its role as a predictor of impaired mobilization.

Previous reports showed worse survival outcomes in patients with this deletion [26,27], however, it is not formally categorized as a high-risk abnormality. The effect of −13 on stem cell mobilization has not been well studied, thus warranting further studies.

### 5.3. High-Risk GEP subtypes and reduced CD34 + HPC collection

Patients classified within the MF and PR subtypes had significantly lower CD34 + HPC yields compared with other GEP subtypes. These subtypes are recognized as “high-risk” and are associated with inferior treatment response and overall survival [16]. To our knowledge, this is the first report of a direct association between MF and PR subtypes and reduced stem cell collection. This observation highlights the aggressive biology of these subgroups and suggests that their adverse behavior may extend beyond treatment resistance to include effects on stem cell mobilization.

### 5.4. Impact of proteasome inhibitor–based induction regimens on stem cell mobilization

During the study period (2017–2020), newly diagnosed MM patients typically received three to four cycles of induction therapy prior to stem cell collection. Following collection, patients may proceed directly to ASCT or continue induction therapy with delayed transplantation at first relapse. Multiple randomized clinical trials have shown superior progression-free survival, among patients undergoing upfront ASCT compared with those receiving conventional therapy alone [12]. Since then, treatment guidelines have changed significantly, especially with the addition of anti-CD38 monoclonal antibodies to first line therapy. In modern era, quadruplet induction protocols (e.g., based on daratumumab) are becoming more popular before ASCT, followed by maintenance therapy, most often using lenalidomide. ASCT remains a standard of care in transplant-eligible patients due to its ability to achieve more durable responses. Therefore, the findings of this study should be interpreted within the context of a pre-quadruplet era, reflecting treatment approaches prior to the widespread adoption of daratumumab-based frontline regimens [28,29].

Over the past two decades, the outcome in MM has improved substantially with the introduction of new agents, including thalidomide, bortezomib, and lenalidomide [12,30]. Bortezomib, lenalidomide, and dexamethasone (collectively known as VRD) were the standard of care for newly diagnosed MM, currently quadruplet induction protocols (e.g. Dara-VRD or Isa-VRD) are used [28,29,31,32]. Other protocols of triplet regimens that include proteasome inhibitors, such as bortezomib- or carfilzomib- based combinations have also gained widespread clinical use [33,34]. Bortezomib and carfilzomib have been shown to enhance stem cell mobilization, likely through modulation of the bone marrow microenvironment and increased chemo-sensitivity of myeloma cells [34,35]. Many studies have demonstrated improved mobilization with bortezomib-based regimens, particularly when combined with G-CSF and cyclophosphamide [36–38]. Conversely, the effect of carfilzomib-based regimens (e.g., KRd) on stem cell collection is more variable. While these regimens may result in more significant hematologic responses, multiple studies have indicated diminished peripheral blood CD34 + HPC counts, lower collection yields, and heightened mobilization requirements in comparison to bortezomib -based regimens (e.g., VRd). These discrepancies may be affected by factors like lenalidomide exposure and the timing of stem cell collection [39].

In our study, a similar pattern was observed across unadjusted analyses, multivariable linear regression, and quantile regression models, which suggests that carfilzomib- and bortezomib-based induction regimens were generally associated with comparable CD34 + HPC mobilization and collection performance. However, quantile regression analysis demonstrated a statistically significant difference at the 25th percentile of first collection yield, indicating that regimen effects may differ among lower mobilizers, whereas no significant differences were observed at the median or higher percentiles. Furthermore, patients receiving both agents did not demonstrate improved mobilization compared with those treated with either agent alone. These findings suggest that while proteasome inhibitor–based induction regimens appear broadly comparable in overall mobilization performance, differential effects may be present in lower-yield subsets, and combined exposure does not appear to provide additive benefit.

This study has some limitations. Despite the large cohort, some genetic subgroups were relatively small, which may have reduced our ability to detect more modest effects on CD34 + HPC yield. In addition, mobilization and treatment practices were not fully uniform over the study period, and this variability may have influenced collection outcomes. Furthermore, additional clinical variables, such ISS stages and comprehensive pre-treatments, were not examined. As this analysis was performed at a single, specialized center, the findings may not be generalizable to all MM populations.

Future work should focus on prospective, multi-center studies to validate these observations, in addition to better understanding the biological mechanisms linking “high-risk” genetic profiles and treatment regimens to impaired stem cell mobilization.

## 6. Conclusion

In this large cohort of patients with multiple myeloma undergoing autologous stem cell mobilization, we examined the influence of induction regimens and underlying cytogenetic features on CD34 + HPC collection. Overall, proteasome inhibitor–based induction strategies demonstrated comparable mobilization outcomes, with no differences observed in adjusted linear or median quantile analyses, although a modest reduction in first collection yield was noted at the lower end of the distribution among carfilzomib-treated patients. In contrast, some molecular subtypes and “high-risk” cytogenetic abnormalities were consistently associated with reduced CD34 + HPC yields. These findings highlight the influence of disease biology, rather than the induction regimen alone, in understanding stem cell mobilization performance. Further research is needed to investigate the possible mechanisms in order to better tailor treatment options.

## Supporting information

S1 Table Multivariable Linear and Quantile Regression Analyses of CD34 + HPC Collection Yield. Quantile regression analyses to assess regimen effects across the distribution of collection outcomes. At the median, carfilzomib-based induction showed no significant difference in first or final CD34 + HPC collection yields, with p-values of 0.32 and 0.63. No significant associations were found at higher quantiles, including the 75th and 90th percentiles.
(DOCX)

S2 Table Impact of common cytogenetic and FISH abnormalities on CD34 + HPC collection yield. Patients with chromosomal abnormalities impact the yield of CD34 + hematopoietic progenitor cells (HPC). A deletion of chromosome 13 had a lower final compared to those without (p = 0.02). For the t(14;16) translocation, patients yielded less on the first collection (p = 0.03), but total yields were similar (p = 0.85). Deletion of chromosome 17 did not show significant differences (p = 0.14 and p = 0.12). Amplification of 1q21 and gain of 14q32 showed reduced total yields (p = 0.04 and p = 0.004), yet first collection yields were not significantly different.
(DOCX)

## References

[1] Bianchi G, Anderson KC. Understanding biology to tackle the disease: Multiple myeloma from bench to bedside, and back. CA Cancer J Clin. 2014;64(6):422–44. doi: 10.3322/caac.21252 25266555

[2] Röllig C, Knop S, Bornhäuser M. Multiple myeloma. Lancet. 2015;385(9983):2197–208. doi: 10.1016/S0140-6736(14)60493-1 25540889

[3] Rajkumar SV, Kumar S. Multiple Myeloma: Diagnosis and Treatment. Mayo Clin Proc. 2016;91(1):101–19. doi: 10.1016/j.mayocp.2015.11.007 26763514 PMC5223450

[4] Chhabra S, Callander N, Watts NL, Costa LJ, Thapa B, Kaufman JL, et al. Stem Cell Mobilization Yields with Daratumumab- and Lenalidomide-Containing Quadruplet Induction Therapy in Newly Diagnosed Multiple Myeloma: Findings from the MASTER and GRIFFIN Trials. Transplant Cell Ther. 2023;29(3):174.e1–174.e10. 10.1016/j.jtct.2022.11.029 36494017

[5] Voorhees PM, Kaufman JL, Laubach J, Sborov DW, Reeves B, Rodriguez C, et al. Daratumumab, lenalidomide, bortezomib, and dexamethasone for transplant-eligible newly diagnosed multiple myeloma: the GRIFFIN trial. Blood. 2020;136(8):936–45. doi: 10.1182/blood.2020005288 32325490 PMC7441167

[6] Callander NS, Silbermann R, Kaufman JL, Godby KN, Laubach J, Schmidt TM, et al. Daratumumab-based quadruplet therapy for transplant-eligible newly diagnosed multiple myeloma with high cytogenetic risk. Blood Cancer J. 2024;14(1):69. doi: 10.1038/s41408-024-01030-w 38649340 PMC11035596

[7] Abdallah NH, Binder M, Rajkumar SV, Greipp PT, Kapoor P, Dispenzieri A, et al. A simple additive staging system for newly diagnosed multiple myeloma. Blood Cancer J. 2022;12(1):21. doi: 10.1038/s41408-022-00611-x 35102148 PMC8803917

[8] Rosenbaum ER, O’Connell B, Cottler-Fox M. Validation of a formula for predicting daily CD34(+) cell collection by leukapheresis. Cytotherapy. 2012;14(4):461–6. doi: 10.3109/14653249.2011.652733 22277012

[9] Pandey S, Cottler-Fox M. Optia® continuous mononuclear collection (CMNC) system is a safe and efficient system for hematopoietic progenitor cells-apheresis (HPC-a) collection and yields a lower product hematocrit (HCT%) than the COBE® spectra system: A retrospective study. J Clin Apher. 2018;33(4):505–13. doi: 10.1002/jca.21629 29603795

[10] Ataca Atilla P, Bakanay Ozturk SM, Demirer T. How to manage poor mobilizers for high dose chemotherapy and autologous stem cell transplantation? Transfus Apher Sci. 2017;56(2):190–8. doi: 10.1016/j.transci.2016.11.005 28034547

[11] Wuchter P, Ran D, Bruckner T, Schmitt T, Witzens-Harig M, Neben K, et al. Poor mobilization of hematopoietic stem cells-definitions, incidence, risk factors, and impact on outcome of autologous transplantation. Biol Blood Marrow Transplant. 2010;16(4):490–9. doi: 10.1016/j.bbmt.2009.11.012 19925876

[12] Kumar S, Giralt S, Stadtmauer EA, Harousseau JL, Palumbo A, Bensinger W, et al. Mobilization in myeloma revisited: IMWG consensus perspectives on stem cell collection following initial therapy with thalidomide-, lenalidomide-, or bortezomib-containing regimens. Blood. 2009;114(9):1729–35. doi: 10.1182/blood-2009-04-205013 19561323

[13] Li S, Fu J, Ma H, Mapara MY, Lentzsch S. Lenalidomide-induced upregulation of CXCR4 in CD34+ hematopoietic cells, a potential mechanism of decreased hematopoietic progenitor mobilization. Leukemia. 2013;27(6):1407–11. doi: 10.1038/leu.2012.323 23138185

[14] Mishra K, Jandial A, Sandal R, Lad D, Prakash G, Khadwal A. Poor Mobilisation After Daratumumab Based Combination Chemotherapy in Patients of Newly Diagnosed Multiple Myeloma. Indian J Hematol Blood Transfus. 2019;35(3):584–6. 31388282 10.1007/s12288-019-01135-4 PMC6646455

[15] Eleutherakis Papaiakovou E, Terpos E, Kanellias N, Migkou M, Gavriatopoulou M, Ntanasis-Stathopoulos I, et al. Impact of daratumumab on stem cell mobilization and collection, engraftment and early post-transplant complications among multiple myeloma patients undergoing autologous stem cell transplantation. Leuk Lymphoma. 2023;64(13):2140–7. doi: 10.1080/10428194.2023.2253479 37655597

[16] Zhan F, Huang Y, Colla S, Stewart JP, Hanamura I, Gupta S, et al. The molecular classification of multiple myeloma. Blood. 2006;108(6):2020–8. doi: 10.1182/blood-2005-11-013458 16728703 PMC1895543

[17] Brioli A, Perrone G, Patriarca F, Pezzi A, Nobile F, Ballerini F, et al. Successful mobilization of PBSCs predicts favorable outcomes in multiple myeloma patients treated with novel agents and autologous transplantation. Bone Marrow Transplant. 2015;50(5):673–8. doi: 10.1038/bmt.2014.322 25642764

[18] Moreb JS, Byrne M, Shugarman I, Zou F, Xiong S, May WS, et al. Poor peripheral blood stem cell mobilization affects long-term outcomes in multiple myeloma patients undergoing autologous stem cell transplantation. J Clin Apher. 2018;33(1):29–37. doi: 10.1002/jca.21556 28556233

[19] Sonneveld P, Avet-Loiseau H, Lonial S, Usmani S, Siegel D, Anderson KC, et al. Treatment of multiple myeloma with high-risk cytogenetics: a consensus of the International Myeloma Working Group. Blood. 2016;127(24):2955–62. doi: 10.1182/blood-2016-01-631200 27002115 PMC4920674

[20] Dewald GW, Kyle RA, Hicks GA, Greipp PR. The clinical significance of cytogenetic studies in 100 patients with multiple myeloma, plasma cell leukemia, or amyloidosis. Blood. 1985;66(2):380–90. doi: 10.1182/blood.v66.2.380.380 3926026

[21] Smadja NV, Bastard C, Brigaudeau C, Leroux D, Fruchart C, Groupe Français de Cytogénétique Hématologique. Hypodiploidy is a major prognostic factor in multiple myeloma. Blood. 2001;98(7):2229–38. doi: 10.1182/blood.v98.7.2229 11568011

[22] Schmidt TM, Fonseca R, Usmani SZ. Chromosome 1q21 abnormalities in multiple myeloma. Blood Cancer J. 2021;11(4):83. doi: 10.1038/s41408-021-00474-8 33927196 PMC8085148

[23] Başcı S, Yiğenoğlu TN, Yaman S, Bozan E, Ulu BU, Bakırtaş M. Does myeloma genetic have an effect on stem cell mobilization? Transfus Apher Sci. 2021;60(6):103249. 34419357 10.1016/j.transci.2021.103249

[24] Lebel E, Lajkosz K, Masih-Khan E, Reece D, Trudel S, Tiedemann R, et al. The Impact of CD34+ Cell Collection Yields for Autologous Transplant on Survival Outcomes in Multiple Myeloma. Clin Lymphoma Myeloma Leuk. 2023;23(11):850–6. doi: 10.1016/j.clml.2023.07.014 37689547

[25] Raschle J, Ratschiller D, Mans S, Mueller BU, Pabst T. High levels of circulating CD34+ cells at autologous stem cell collection are associated with favourable prognosis in multiple myeloma. Br J Cancer. 2011;105(7):970–4. doi: 10.1038/bjc.2011.329 21878938 PMC3185945

[26] Binder M, Rajkumar SV, Ketterling RP, Greipp PT, Dispenzieri A, Lacy MQ, et al. Prognostic implications of abnormalities of chromosome 13 and the presence of multiple cytogenetic high-risk abnormalities in newly diagnosed multiple myeloma. Blood Cancer J. 2017;7(9):e600. doi: 10.1038/bcj.2017.83 28862698 PMC5709752

[27] Fassas AB-T, Spencer T, Sawyer J, Zangari M, Lee C-K, Anaissie E, et al. Both hypodiploidy and deletion of chromosome 13 independently confer poor prognosis in multiple myeloma. Br J Haematol. 2002;118(4):1041–7. doi: 10.1046/j.1365-2141.2002.03757.x 12199783

[28] Richardson PG, Jacobus SJ, Weller EA, Hassoun H, Lonial S, Raje NS. Triplet therapy, transplantation, and maintenance until progression in myeloma. N Engl J Med. 2022;387(2):132–47. 35660812 10.1056/NEJMoa2204925 PMC10040899

[29] Kumar SK, Callander NS, Adekola K, Anderson LD, Baljevic M, Baz R. NCCN Guidelines® Insights: Multiple Myeloma, Version 1.2025. J Natl Compr Canc Netw. 2025;23(5):132–40. 40340857 10.6004/jnccn.2025.0023

[30] Rajkumar SV. Multiple myeloma: 2022 update on diagnosis, risk stratification, and management. Am J Hematol. 2022;97(8):1086–107. doi: 10.1002/ajh.26590 35560063 PMC9387011

[31] Durie BGM, Hoering A, Abidi MH, Rajkumar SV, Epstein J, Kahanic SP, et al. Bortezomib with lenalidomide and dexamethasone versus lenalidomide and dexamethasone alone in patients with newly diagnosed myeloma without intent for immediate autologous stem-cell transplant (SWOG S0777): a randomised, open-label, phase 3 trial. Lancet. 2017;389(10068):519–27. doi: 10.1016/S0140-6736(16)31594-X 28017406 PMC5546834

[32] Rajkumar SV, Kumar S. Multiple myeloma current treatment algorithms. Blood Cancer J. 2020;10(9):94. doi: 10.1038/s41408-020-00359-2 32989217 PMC7523011

[33] Kapoor P, Ramakrishnan V, Rajkumar SV. Bortezomib combination therapy in multiple myeloma. Semin Hematol. 2012;49(3):228–42. doi: 10.1053/j.seminhematol.2012.04.010 22726546 PMC3597231

[34] Groen K, van de Donk N, Stege C, Zweegman S, Nijhof IS. Carfilzomib for relapsed and refractory multiple myeloma. Cancer Manag Res. 2019;11:2663–75. doi: 10.2147/CMAR.S150653 31037034 PMC6450182

[35] Ghobadi A, Rettig MP, Cooper ML, Holt MS, Ritchey JK, Eissenberg L, et al. Bortezomib is a rapid mobilizer of hematopoietic stem cells in mice via modulation of the VCAM-1/VLA-4 axis. Blood. 2014;124(17):2752–4. doi: 10.1182/blood-2014-08-595967 25342668 PMC4208289

[36] Mirgh S, Bagal B, Punatar S, Gokarn A, Jindal N, Chichra A, et al. Moving Beyond G-CSF Mobilization-Learning From a 15-Year Experience of Different Stem Cell Mobilization Regimens in Multiple Myeloma. Cancer Med. 2025;14(14):e71068. doi: 10.1002/cam4.71068 40667650 PMC12264575

[37] Bagal B, Gokarn A, Punatar S, Das S, Bonda A, Nayak L, et al. Bortezomib and cyclophosphamide based chemo-mobilization in multiple myeloma. Int J Hematol. 2020;112(6):835–40. doi: 10.1007/s12185-020-02973-z 32876851

[38] Nishimura KK, Barlogie B, van Rhee F, Zangari M, Walker BA, Rosenthal A, et al. Long-term outcomes after autologous stem cell transplantation for multiple myeloma. Blood Adv. 2020;4(2):422–31. doi: 10.1182/bloodadvances.2019000524 31990333 PMC6988393

[39] Bal S, Landau HJ, Shah GL, Scordo M, Dahi P, Lahoud OB, et al. Stem Cell Mobilization and Autograft Minimal Residual Disease Negativity with Novel Induction Regimens in Multiple Myeloma. Biol Blood Marrow Transplant. 2020;26(8):1394–401. doi: 10.1016/j.bbmt.2020.04.011 32442725 PMC7371503

