# Supplementary material for: Collection of autologous CD34+ hematopoietic progenitor cells (HPC) in multiple myeloma: CD34 + cell collection yield in relation to molecular subtype, karyotype, and FISH results
Source: PLoS One. 2026 May 12;21(5):e0349212. doi: 10.1371/journal.pone.0349212 (PMC13166896; doi:10.1371/journal.pone.0349212)
Supplement: S1 Table — Quantile regression analyses to assess regimen effects across the distribution of collection outcomes. At the median, carfilzomib-based induction showed no significant difference in first or final CD34 + HPC collection yields, with p-values of 0.32 and 0.63. No significant associations were found at higher quantiles, including the 75th and 90th percentiles. (DOCX) [file pone.0349212.s001.docx]

S1 Table: Multivariable Linear and Quantile Regression Analyses of CD34+ HPC Collection Yield

| **Outcome** | **Model** | **Quantile** | **β Coefficient (×10⁶/kg)** | **95% Confidence Interval** | **p-value** |
| --- | --- | --- | --- | --- | --- |
| **First collection CD34+ HPC yield** | Linear regression | — | +0.38 | −3.13 to 3.90 | 0.83 |
|  | Quantile regression | 25th percentile | −2.94 | −5.73 to −0.15 | 0.040 |
|  |  | 50th percentile (median) | −2.52 | −7.51 to 2.48 | 0.323 |
|  |  | 75th percentile | +1.08 | −8.08 to 10.23 | 0.818 |
|  |  | 90th percentile | +7.32 | −6.00 to 20.65 | 0.282 |
| **Final cumulative CD34+ HPC yield** | Linear regression | — | +1.83 | −2.47 to 6.12 | 0.40 |
|  | Quantile regression | 25th percentile | −2.53 | −5.54 to 0.48 | 0.100 |
|  |  | 50th percentile (median) | −1.38 | −6.91 to 4.16 | 0.626 |
|  |  | 75th percentile | +5.31 | −6.30 to 16.92 | 0.370 |
|  |  | 90th percentile | +8.47 | −2.86 to 19.80 | 0.144 |
