# Supplementary material for: Collection of autologous CD34+ hematopoietic progenitor cells (HPC) in multiple myeloma: CD34 + cell collection yield in relation to molecular subtype, karyotype, and FISH results
Source: PLoS One. 2026 May 12;21(5):e0349212. doi: 10.1371/journal.pone.0349212 (PMC13166896; doi:10.1371/journal.pone.0349212)
Supplement: S2 Table — Patients with chromosomal abnormalities impact the yield of CD34 + hematopoietic progenitor cells (HPC). A deletion of chromosome 13 had a lower final compared to those without (p = 0.02). For the t(14;16) translocation, patients yielded less on the first collection (p = 0.03), but total yields were similar (p = 0.85). Deletion of chromosome 17 did not show significant differences (p = 0.14 and p = 0.12). Amplification of 1q21 and gain of 14q32 showed reduced total yields (p = 0.04 and p = 0.004), yet first collection yields were not significantly different. (DOCX) [file pone.0349212.s002.docx]

S2 Table: Impact of common cytogenetic and FISH abnormalities on CD34+ HPC collection yield

| Mutation | p-value (First Collection Yield) | Normal Count (First) (x 10^6^/kg) | Abnormal Count (First) (x 10^6^/kg) | Normal Mean (First) (x 10^6^/kg) | Abnormal Mean (First) (x 10^6^/kg) | p-value (Final CD34 Yield) | Normal Count (Final) (x 10^6^/kg) | Abnormal Count (Final) (x 10^6^/kg) | Normal Mean (Final) (x 10^6^/kg) | Abnormal Mean (Final) (x 10^6^/kg) |
| --- | --- | --- | --- | --- | --- | --- | --- | --- | --- | --- |
| *del(1p32)* | 0.67 | 217 | 32 | 14.52 | 15.50 | 0.73 | 217 | 32 | 26.36 | 27.32 |
| *amp(1q21)* | 0.17 | 3 | 29 | 19.50 | 10.85 | 0.04 | 3 | 29 | 40.47 | 23.21 |
| *del(13q14.3)* | 0.57 | 225 | 24 | 14.87 | 13.36 | 0.70 | 225 | 24 | 26.78 | 25.56 |
| *-13* | 0.08 | 168 | 81 | 15.93 | 12.98 | 0.02 | 168 | 81 | 28.55 | 23.98 |
| *del(17p13.1)* | 0.23 | 218 | 32 | 15.32 | 12.43 | 0.28 | 218 | 32 | 27.27 | 24.21 |
| *t(4;14)(p16.3;q32)* | 0.47 | 107 | 21 | 13.78 | 15.89 | 0.49 | 107 | 21 | 25.10 | 27.16 |
| *-17* | 0.14 | 209 | 9 | 14.97 | 8.54 | 0.12 | 209 | 9 | 27.19 | 19.32 |
| *t(11;14)(q13;q32)* | 0.94 | 74 | 45 | 12.83 | 12.97 | 0.90 | 74 | 45 | 24.25 | 23.96 |
| *+14q32* | 0.11 | 4 | 25 | 28.13 | 13.00 | 0.004 | 4 | 25 | 55.90 | 23.75 |
| *t(14q32)* | 0.78 | 98 | 13 | 14.70 | 15.64 | 0.92 | 98 | 13 | 26.99 | 26.55 |
| *t(14;20)(q32;q12)* | 0.33 | 134 | 2 | 13.72 | 5.25 | 0.74 | 134 | 2 | 25.14 | 22.10 |
| *+20q12* | 0.62 | 2 | 30 | 16.30 | 12.09 | 0.75 | 2 | 30 | 21.60 | 24.62 |
| *del(14q32)* | 0.85 | 3 | 57 | 18.23 | 16.75 | 0.70 | 3 | 57 | 24.33 | 28.30 |
| *17* | 0.10 | 2 | 28 | 24.15 | 11.48 | 0.55 | 2 | 28 | 33.60 | 26.27 |
| *t(14;16)(q32;q23)* | 0.03 | 110 | 12 | 14.43 | 6.23 | 0.85 | 110 | 12 | 25.16 | 24.38 |
| *dup(1q21)* | 0.13 | 152 | 70 | 16.10 | 13.35 | 0.27 | 152 | 70 | 27.84 | 25.44 |
